# Beyond a single system: Lessons from Ghana's discontinued national EHR and the imperative for an interoperable digital health ecosystem

**DOI:** 10.4314/gmj.v59i4.1

**Published:** 2025-12

**Authors:** Yaw Adjei Ofori-Adjei

**Affiliations:** Department of Medicine & Therapeutics, Korle Bu Teaching Hospital, Accra, Ghana

**Keywords:** Electronic Medical Records, Digital Health Interoperability, LHIMS / Ghana Health Information Systems, National eHealth Strategy, Health Information Exchange (HIE)

Ghana's pursuit of a national electronic health record (EHR) system dates back over a decade before the introduction of the Lightwave Health Information Management System (LHIMS). In 2010, the Ministry of Health launched the National e-Health Strategy, which explicitly called for the application of information and communication technologies to improve health-system performance and service efficiency.^1^ The strategy identified the lack of interoperability, fragmented hospital data silos, and manual patient records as critical barriers. It therefore proposes a national e-health architecture grounded in shared standards and enhanced coordination between public and private providers. It also emphasised that early e-health initiatives were small, uncoordinated pilots and highlighted the need for a unified national platform for patient information.^1^

Following this policy groundwork, the Magnum Hospital Information System (HIS) was introduced around 2012 as part of an e-Health pilot at Korle-Bu Teaching Hospital, Wa Regional Hospital, and Zebilla District Hospital. Implemented through a partnership between a Ghanaian ICT Company (IPMC) and an Indian company (Healthfore), the Magnum HIS was supposed to be a comprehensive EHR platform designed to automate clinical, financial, and administrative processes within hospitals. It incorporated core modules such as patient registration, appointments, electronic health records, laboratory and pharmacy systems, and billing, and conformed to international standards, including ICD-10 and HL7, for interoperability.^2^ This initiative represented Ghana's first structured attempt at deploying a multi-site electronic health record system under a government-backed e-health pilot.

Lessons from this pilot—particularly the high infrastructure demands, limited internet connectivity in regional hospitals, and the need for sustained local technical capacity—directly informed the subsequent development of the Lightwave Health Information Management System (LHIMS). LHIMS emerged in 2017 as a locally engineered successor intended to provide a unified national EHR platform, beginning with its rollout at Cape Coast Teaching Hospital and subsequently across multiple Ghana Health Service facilities^3^ Thus, LHIMS built upon the vision articulated in the 2010 National e-Health Strategy and the earlier Magnum HIS project, marking Ghana's most ambitious national step toward digital health integration. The LHIMS contract with Ghana's Ministry of Health, initiated in 2016 and subsequently entered phase II in March 2019, expired in December 2024. According to Lightwave EHealthCare Solutions, at the time of contract expiration, LHIMS had been deployed to 253 major health facilities, namely four (4) Teaching Hospitals, six (6) Regional Hospitals, and two hundred and forty-three (243) District (and other) Hospitals.^4^

Following the expiration of the LHIMS contract and a subsequent period of public controversy surrounding the platform's performance and governance, the Ministry of Health formally announced its discontinuation. It introduced a new, state-owned digital platform, the Ghana Health Information Management System (GHIMS)^5^ The decision to discontinue LHIMS was driven by a series of interlinked failures, including the inability to digitise the targeted 950 health facilities despite several contract extensions, deficient hardware procurement, and the LHIMS cloud infrastructure reportedly located in India, raising national concerns about the control of health data. The vendor of LHIMS has refuted some of these claims in their press release of 30th October 2025.^4^,^6^

The recent discontinuation of Ghana's LHIMS presents a critical inflection point for digital health in the country. While the vision to digitise healthcare was commendable, the project's failure offers a stark lesson in the perils of overlooking established global best practices for health information system design, governance, and interoperability.^7^ This experience is not isolated; it echoes similar setbacks in other nations and provides a compelling case for a fundamental policy shift.^8^

The LHIMS was developed organically as a centralised national electronic health record (EHR) without adhering to global data exchange standards like HL7 FHIR (Fast Healthcare Interoperability Resources).^9^ This created immediate interoperability barriers, resulting in data silos and preventing seamless communication with other platforms.^10^ Reports from hospitals nationwide indicated that health workers found the system unintuitive, leading to workarounds, incomplete data entry, and ultimately, compromised data quality that undermines clinical decision-making and public health surveillance.^11^

A critical flaw was the mandatory, one-size-fits-all rollout to all public health institutions, irrespective of their infrastructure, size, or digital readiness. Facilities, particularly in remote areas, faced unreliable internet access, inadequate training, and limited technical support.^12^

When system outages^13^ or payment disputes with the vendor occurred—as reported in the Ashanti Region and elsewhere—hospitals were forced to revert to paper, disrupting care continuity and highlighting the system's fragility.^14^,^15^

The core policy misstep was the centralised, non-competitive procurement model. This approach conflates digital sovereignty with state-owned software, a misconception that has hampered similar initiatives globally. For instance, South Africa's initial attempts at a monolithic, state-developed system faced parallel challenges of low adoption and scalability before moving toward a standards-based framework.^16^,^17^ The United Kingdom's National Programme for IT (NPfIT), launched in 2002 with an initial budget of £6.2 billion, suffered a similar fate.^18^ In contrast, successful digital health nations like Estonia and Kenya^19^ have adopted a different paradigm. Their ministries of health focus on defining national health information exchange (HIE) standards and governance, while allowing a competitive market of private vendors to develop systems that comply with these standards.^20^ This fosters innovation, adaptability, and long-term sustainability.

A powerful parallel exists in the financial sector. The development of standardised payment protocols like Visa/Mastercard or mobile money interoperability frameworks (e.g., Ghana's GhIPSS) did not require governments to build their own proprietary banking platforms. Instead, by setting technical and security standards, regulators enabled a vibrant ecosystem of private banks and fintech companies to innovate. This competition dramatically broadened financial access and inclusivity, from mobile money in Kenya to digital banking in India. The health sector can achieve similar gains by focusing on the “rails” of data exchange (the standards) rather than the “trains” (the specific EHR applications).

The LHIMS experience demonstrates that a sustainable national digital health architecture depends on open standards, modular design, and collaborative governance, not on a single proprietary system.^6^ The limited consultation with end-users—clinicians, nurses, and health information officers—further eroded trust and adoption.^21^

Moving forward, Ghana's Ministry of Health has a valuable opportunity to re-strategise. The priority must be the development of a National Digital Health Interoperability Framework, aligned with WHO guidelines^22^ and architectures like OpenHIE.^23^,^24^ This framework should:

1. Mandate core interoperability standards (HL7 FHIR, ICD-11, SNOMED CT) for all health data systems.^22^
2. Foster a competitive EHR vendor ecosystem by accrediting multiple systems that comply with national standards.
3. Establish robust governance for data stewardship, patient privacy, and cybersecurity.^25^
4. Promote public-private partnerships for infrastructure, innovation, and capacity building.
5. Prioritise user-centred design and phased implementation to ensure usability and ownership.^26^

Ghana's ambition for a digital health transformation remains urgent and achievable. However, the lesson from LHIMS is clear: sustainable success requires a standards-driven, participatory, and modular approach. By learning from both the failures of centralised models and the successes of interoperable ecosystems in health and finance, Ghana can build a resilient, patient-centred health information system fit for the future.
